# Association between Smoking and Health Outcomes in Postmenopausal Women Living with Multiple Sclerosis

**DOI:** 10.1155/2014/686045

**Published:** 2014-04-22

**Authors:** Rachel Jawahar, Unsong Oh, Charles Eaton, Nicole Wright, Hilary Tindle, Kate L. Lapane

**Affiliations:** ^1^Department of Epidemiology and Community Health, Virginia Commonwealth University School, Richmond, VA 23284, USA; ^2^Department of Neurology, Virginia Commonwealth University School, Richmond, VA 23284, USA; ^3^Center for Primary Care and Prevention, Memorial Hospital of Rhode Island, Pawtucket, RI 02861, USA; ^4^Department of Epidemiology, University of Alabama Birmingham, Birmingham, AL 35294, USA; ^5^Department of Medicine, University of Pittsburgh, Pittsburgh, PA 15260, USA; ^6^Department of Quantitative Health Sciences, University of Massachusetts Medical School, 55 Lake Avenue North, Worcester, MA 01655, USA

## Abstract

*Background*. In multiple sclerosis (MS), symptom management and improved health-related quality of life (HrQOL) may be modified by smoking. *Objective*. To evaluate the extent to which smoking is associated with worsened health outcomes and HrQOL for postmenopausal women with MS. *Methods*. We identified 251 Women's Health Initiative Observational Study participants with a self-reported MS diagnosis. Using a linear model, we estimated changes from baseline to 3 years for activities of daily living, total metabolic equivalent tasks (MET) hours per week, mental and physical component scales (MCS, PCS) of the SF-36, and menopausal symptoms adjusting for years since menopause and other confounders. *Results*. Nine percent were current and 50% past smokers. Age at smoking initiation was associated with significant changes in MCS during menopause. PCS scores were unchanged. While women who had ever smoked experienced an increase in physical activity during menopause, the physical activity levels of women who never smoked declined. Residual confounding may explain this finding. Smoking was not associated with change in menopausal symptoms during the 3-year follow-up. *Conclusion*. Smoking was not associated with health outcomes among post-menopausal women with MS.

## 1. Introduction

Multiple sclerosis (MS) is a chronic disease of the central nervous system with symptoms that impact health-related quality of life (HrQOL) [1, 2]. MS disproportionately affects more women than men [3]. The extent to which the menopausal transition in women worsens MS symptoms remains largely unexplored. Previous studies have addressed symptom management in MS through modifiable risk factors such as smoking [4]. Forty percent [5, 6] of women with MS are current smokers, yet how smoking affects MS outcomes after menopause is unknown.

In both menopausal and postmenopausal women, current smokers report increased odds of vasomotor symptoms, hot flashes, forgetfulness [7], and worsened HrQOL [8]. In MS, smoking has been linked to increased incidence [6] and faster MS progression [5, 9] leading to worse health outcomes [10]. More than 37 million women are approaching or experiencing menopause [11] in the aging U.S. population [12]. This underscores the need for greater focus on symptom management for women with MS during the menopausal transition and beyond. Using a multicenter prospective study of U.S. postmenopausal women, we aimed to evaluate the extent to which health outcomes including health-related quality of life and indicators of physical functioning are worsened for women with MS who currently smoke or previously smoked relative to women with MS who never smoked.

## 2. Materials and Methods

The Virginia Commonwealth University Institutional Review Board approved this study.

### 2.1. Participants

The Women's Health Initiative Observational Study (WHI-OS), sponsored by the National Institutes of Health and the National Heart, Lung, and Blood Institute, followed 93,676 racially diverse women aged 50 to 79 years and recruited from 40 clinical centers throughout the U.S. [13]. Women were eligible for participation in the WHI-OS if they were postmenopausal, not enrolled in other WHI clinical trials, and unlikely to relocate or die within 3 years. Protocols for WHI-OS were reviewed and approved by human subjects review committees at participating institutions [14]. Participants for the current study were included if they reported “yes” to the question “Has a doctor ever told you you had MS?” Analyses included 251 WHI-OS participants who completed baseline and year three assessments and had complete information on smoking history.

### 2.2. Determination of Smoking Status

Smoking status was determined at baseline using the following questions: “During your entire life, have you smoked at least 100 cigarettes?” and “Do you smoke cigarettes now?” The responses to these questions were combined to classify women as current, past, or never smokers. For women who reported ever having smoked, we classified women according to their age at smoking initiation (<20 years, 20–24 years, and ≥25 years), the number of cigarettes smoked per day (<15/day, ≥15/day), and the number of years smoked regularly (<30, ≥30 years). Past smokers were asked age at cessation and classified as <40 years, ≥40 years. Calculated by the WHI Clinical Coordinating Center, the number of smoking pack-years [15] was categorized as <10, 10–29, and ≥30 pack-years.

### 2.3. Outcome Ascertainment

We evaluated HrQOL, menopausal symptoms, and indicators of physical functioning and activity measures. HrQOL was measured using the RAND 36-item health survey (SF-36) [16] which has been validated in MS patients [17]. We calculated the mental component score (MCS) and the physical component score (PCS), each ranging from 0 (lower health) to 100 points (better health) with 50 representing the mean score in the general population. Activities of daily living (ADLs) (modified from the original Katz et al. [18] index) consisted of four separate items regarding ability to eat, ability to get in and out of bed, dress, and/or take a bath on her own. Each item had three possible values (1 = without help, 2 = some help, and 3 = completely unable). For baseline and year 3, scores (ranging from 4 to 12) were summed to represent overall ADLs with a lower score indicating better health. Baseline ADL scores were subtracted from year 3 ADL scores so that a positive change score represented a decline in ADLs. Physical activity was computed from self-reported energy expenditures for recreational activities, including walking and other mild/moderate/strenuous activities (total metabolic equivalent tasks (MET) hours/week). Calculated MET hours per week are comparable to physical activity diaries [19]. Baseline scores were subtracted from year 3 scores such that a positive change score indicated an increase in physical activity.

Our focus was on menopausal symptoms, rather than neurological symptoms. Based on the Postmenopausal Estrogen/Progestin Interventions symptom tool [20], we considered the following items as menopausal symptoms: forgetfulness, difficulty in concentrating, mood swings, joint pain or stiffness, headaches or migraines, breast tenderness, increased or decreased appetite, hot flashes, night sweats, vaginal/genital irritation, and vaginal/genital dryness. Participants were asked how bothersome each symptom was in the past four weeks (0 = did not occur, 1 = mild, 2 = moderate, and 3 = severe). For each symptom, we created a binary variable coded as 0 = did not occur and 1 = symptom occurred (mild, moderate, and severe). At baseline and year 3, a summary measure was constructed by adding the number of symptoms reported (minimum score possible: 0; maximum score possible: 12). We treated the outcome as a continuous variable by subtracting the baseline sum from year 3 summary measure.

### 2.4. Potential Confounders

Potential confounders considered included age, education, race/ethnicity, years since menopause, alcohol use, body mass index (BMI), depression, menopausal hormone therapy (MHT) use, and vitamin D intake. Years since menopause were calculated as the difference between the youngest reported age when menses ceased (age when participant experienced last menstruation, oophorectomy, or initiated MHT) and age at baseline. Baseline alcohol use was assessed using the Food Frequency Questionnaire (FFQ) and categorized as never, past, and current use. BMI was calculated in kg/m^2^ units from heights and weights measured with calibrated balances and stadiometers (<18.5 kg/m^2^, 18.5 kg/m^2^, and < 25 kg/m^2^; 25 kg/m^2^ to 30 kg/m^2^; and ≥ 30 kg/m^2^). MHT (unopposed estrogen and/or estrogen plus progesterone) was classified as current, past, or never use. Using FFQ and supplement use, vitamin D insufficiency was defined as <400 IU [21], as this was the standard used at the time of the study. The CES-D [22] was used to evaluate the depression status and participants with scores above 16 were considered to have clinical levels of depression.

### 2.5. Statistical Analysis

We reported the sociodemographic, clinical, and smoking characteristics by smoking status. Multivariable linear models estimated associations between differences in 3-year HrQOL, ADL, and physical activity scores and number of menopausal symptoms by baseline smoking status. We examined univariate distributions of each score's differences and years since menopause to ensure normality. Multicollinearity was ruled out by evaluating correlations between each potential confounder. We visually inspected residual plots, Q-Q plots, and studentized residuals. Beta coefficients and corresponding 95% confidence intervals (CI) were derived from the adjusted models and corrected for the number of outcomes evaluated in the study using Bonferroni's method.

## 3. Results

Of the 251 women with MS, 6% changed smoking status from baseline measures to year 3. Nearly 9% of women were current smokers (*n* = 23) and 50.2% were past smokers (*n* = 126) (Table 1). Current smokers were younger than past smokers. While 64.7% of never smokers reported ever using alcohol, 82.6% of current and 80.2% of past smokers reported current alcohol use. Depression varied by smoking status with 9.1% of current smokers, 20.8% of former smokers, and 25.0% of those who never smoked experiencing depression at baseline. All women, regardless of smoking status, had less than 800 IU vitamin D intake per day from food, over the counter supplements, and/or prescribed supplements. There were no current smokers who had less than 400 IU vitamin D; 3.2% and 4.9% of past and never smokers, respectively, had levels below this threshold. Most women began smoking at 25 years of age or older and most reported regularly smoking less than 15 cigarettes per day (Table 1).

Table 2 shows the changes in HrQOL from baseline to year three of follow-up by smoking status. Age at smoking initiation was associated with significant changes in MCS during menopause in women with MS. PCS scores were unchanged. No differences in change in MCS scores were observed based on overall smoking status (current versus past versus never smoker). Smoking pack-years were not associated with changes in PCS or MCS. Past smokers who reported quitting at the age of 40 or older had lower MCS scores (adjusted *β*: −5.21, 95% CI: −9.3 to −1.1).

Table 3 shows the association between various definitions of smoking and change in ADLs and physical activity from baseline to year 3. None of the associations between ADL change and smoking were statistically significant. Having ever smoked was associated with changes in physical activity. While all women's physical activity declined, current and former smokers were less likely to have reported reductions in their physical activity relative to nonsmokers. For example, women who reported never smoking experienced a decrease of 3.66 MET task hours per week in physical activity. Former smokers experienced a decline of 0.60 MET task hours and current smokers a decline of 0.19 MET task hours. Relative to women who never smoked, we observed a slower decline in physical activity for former smokers (adjusted *β*: 3.76, 95% CI: 0.00 to 7.6).

The five most prevalent menopausal symptoms reported at baseline included joint pain/stiffness (74%), forgetfulness (68%), difficulty in concentrating (48%), headaches (45%), mood swings (42%), and vaginal dryness (31%). Aside from joint pain (10%), few ranked symptoms as severe. Differences in change in menopausal symptoms by overall smoking status were not observed (Table 4). Changes in menopausal symptoms were minimal over the 3-year period. None of the variables categorizing smoking status were associated with changes in menopausal symptoms.

## 4. Conclusions

To our knowledge, this is the first study to estimate the association between smoking status and outcomes in postmenopausal women with MS. Although the WHI-OS followed nearly 100,000 women, only a few hundred women noted that a physician had told them they had MS. Nearly half of these women were past smokers, and most had ceased at the age of 40 or older. Women with MS who began smoking at a young age had worse mental HrQOL at year 3 than at baseline, indicating a decline in cognition during menopause for those who began smoking at the age of 20 or younger. Little change was observed in ADLs regardless of smoking status. While never smokers reported a reduction in physical activity from baseline to year 3, the reductions in physical activity reported by current and former smokers were less marked. The study included all postmenopausal women even though most women experience menopausal symptoms between the ages of 50 to 59. This may explain why we did not observe significant changes in reports of menopausal symptoms within the 3-year observation period. Nevertheless, smoking status was not associated with changes in menopausal symptoms.

Estimates of prevalence of smoking are much lower in this study than in previous reports of 40% [5]. We found that only 9% of women reported current smoking and 59% reported that they had ever smoked. Our data are consistent with previous findings reporting 6.3% current smokers in the WHI-OS [23]. The findings more likely reflect the strong decline in smoking prevalence observed in both men and women [24]. Previous studies have linked smoking history to worse outcomes in the MS disease process [5]. Thus, the possibility of survival bias must be considered when interpreting these findings. Changes in HrQOL and physical functioning and activity measures may be more strongly related to current smoking than past smoking. These data must be considered in light of the unique sample. Decreases in motor function have been noted for MS patients within 10 minutes of smoking a cigarette [25]. Current smokers were less likely to engage in more intense physical activity over eight years of follow-up [26]. The patterns of changes in physical activity by smoking status may suggest that current smokers compensate for smoking by maintaining physical activity. This finding must be confirmed in studies with a larger sample size. It is also possible that this association is confounded by markers of disease severity in MS or residual confounding by comorbid conditions. Lastly, survival bias cannot be ruled out.

Smoking has been associated with increases in MS incidence through its alterations to the blood-brain barrier [27] by nitric oxide [28]. Younger age at smoking initiation has been shown to be associated with an increased risk of MS [29] and worsened prognosis from disease onset [30]. In our study, younger age at smoking initiation was associated with decrease in mental HrQOL. Because women who reported ever having smoked may be more likely to develop progressive disease than never smokers [5], smoking cessation at older ages may be too late to improve mental HrQOL in postmenopause. Regardless of our specific findings, counseling women with MS to quit smoking at the earliest age possible is prudent.

The strengths of this study include the diverse outcomes available in the WHI-OS. While no MS-specific measures were collected, many of the outcomes measured (e.g., SF-36 scales, ADLs) are included in MS-specific composite measures. The WHI-OS provides data not always present in MS registries, such as specific questions regarding menopausal symptoms and the severity of the symptoms. Further, the WHI included detailed information on the frequency and duration of smoking (e.g., number of cigarettes per day, years of smoking before cessation) as well as an array of potentially confounding factors such as sociodemographics, BMI, and vitamin D intake.

Several limitations must be kept in mind. The sample size is small. Some may question the participant-reported physician diagnoses of MS used in the WHI. A validation study of self-reported diagnosis of MS in the North American Research Committee on MS registry showed a 98.79% sensitivity of self-report when compared to chart review and/or physician report [31]. Thus, we were comfortable with our decision to use the self-reported diagnosis of MS available in the WHI-OS.

This study evaluated the effects of smoking on HrQOL and physical measures in postmenopausal women with MS. Despite the large size of the WHI-OS, we had a relatively small sample of women with MS in our study. As these women were healthier than the general population and few were current smokers, effects were not found for all outcomes by all smoking frequencies or duration. Nevertheless, women with MS should be encouraged to quit smoking at the earliest age possible. Patterns were found pointing to an association between initiation of smoking at younger ages and decline of mental HrQOL during menopause. Longitudinal studies of age at smoking initiation and relevant outcomes for older women with MS are needed.

## Figures and Tables

**Table 1:** Baseline characteristics of postmenopausal women with multiple sclerosis by smoking status in the Women's Health Initiative Observational Study.

Baseline characteristics	Current smoker (*n* = 23)	Past smoker (*n* = 126)	Never smoker (*n* = 102)
	Median (standard deviation)		
Years since menopause	11.9 (7.2)	13.2 (8.4)	13.8 (9.4)

	Percentages		
Age			
<50–59 years	65.2	48.4	46.1
60–69 years	34.8	40.5	37.3
70–79+ years	0	11.1	16.7
Race/ethnicity			
Non-Hispanic white	89.5	90.0	90.5
Non-Hispanic black	10.5	6.4	2.4
Hispanic	0	3.6	3.6
Education			
≤High school	13.0	16.0	16.8
Some college	47.8	36.0	36.6
≥College graduate	39.1	48.0	46.5
Have any health insurance	100	98.4	95.1
Depressed	9.1	20.8	25.0
Body mass index (kg/m^2^)			
<18.5 (underweight)	8.7	4.8	7.8
18.5 to <25 (normal)	47.8	48.4	42.2
25 to <30 (overweight)	26.1	26.2	33.3
30+ (obese)	17.4	20.6	16.7
Alcohol use			
Never drinker	0	4.0	18.6
Past drinker	17.4	15.9	16.7
Current drinker	82.6	80.2	64.7
Menopause hormone therapy			
Never user	34.8	34.1	35.3
Past user	8.7	18.3	12.8
Current user	56.5	47.6	52.0
Vitamin D < 400 IU per day	0	3.2	4.9
Age started smoking (years)			
<20	16.1	12.0	N/A
20 to 24	30.1	31.0	N/A
25 or older	53.8	57.1	N/A
Cigarettes smoked (per day)			
<15	52.0	55.1	N/A
15 or more	48.0	44.9	N/A
Years smoked regularly			
<10	4.3	27.3	N/A
10 to 29	20.2	47.6	N/A
30 to 49	63.4	23.7	N/A
50 and more	12.0	1.4	N/A
Pack-years			
<10	22.2	44.9	N/A
10 to 29	35.1	31.7	N/A
30 to 49	29.2	14.5	N/A
50 or more	13.6	8.9	N/A
Age quit smoking (years)			
<20	N/A	1.8	N/A
20 to 29	N/A	19.2	N/A
30 to 39	N/A	25.2	N/A
40 or older	N/A	53.8	N/A

**Table 2:** Association between smoking status and change in health-related quality of life measures over 3 years among postmenopausal women with multiple sclerosis in the Women's Health Initiative Observational Study.

Exposure	ΔPhysical component score (3-year baseline)			ΔMental component score (3-year baseline)		
	Mean change (standard deviation)	*β*-Coefficient		Mean change (standard deviation)	*β*-Coefficient	
		Crude	Adjusted^1^ (95% confidence interval)		Crude	Adjusted^1^ (95% confidence interval)
Smoking history						
Never smokers	−0.65 (8.6)	Ref.	Ref	−0.84 (12.1)	Ref.	Ref.
Ever smokers	−1.10 (9.5)	−0.45	0.05 (−3.3 to 3.4)	0.12 (11.5)	0.96	0.25 (−4.1 to 4.6)
Former smokers	−0.74 (9.1)	−0.10	1.67 (−4.0 to 7.3)	−0.25 (11.2)	0.59	−1.82 (−5.9 to 9.5)
Current smokers	−2.97 (11.7)	−2.33	−0.90 (−6.7 to 4.9)	2.08 (13.4)	2.91	1.80 (−9.3 to 5.6)
Age started smoking (years)						
<20	−1.25 (9.6)	1.25	1.64 (−7.0 to 10.3)	−0.93 (11.4)	−10.03	−10.45 (−19.1 to −1.8)
20 to 24	−0.58 (9.0)	1.93	2.33 (−6.9 to 11.6)	0.72 (11.2)	−8.37	−8.9 (−18.2 to 0.3)
25 or older	−2.51 (12.3)	Ref.	Ref.	9.09 (12.4)	Ref.	Ref.
Cigarettes smoked (per day)						
<15	−1.73 (8.3)	Ref.	Ref.	0.71 (10.3)	Ref.	Ref.
15 or more	−0.46 (10.7)	1.64	1.32 (−2.9 to 5.5)	−0.47 (12.7)	−1.07	−1.16 (−6.7 to 4.4)
Years smoked regularly						
<30	−1.89 (8.8)	Ref.	Ref.	1.12 (10.6)	Ref.	Ref.
30 or more	−0.12 (11.1)	1.77	2.41 (−2.0 to 6.8)	−1.89 (13.3)	−3.01	−3.29 (−9.1 to 2.6)
Number of smoking pack-years						
<10	−1.65 (8.2)	Ref.	Ref.	−0.05 (11.2)	Ref.	Ref.
10 to 29	−2.31 (9.0)	−0.14	−1.55 (−6.0 to 2.9)	2.89 (10.1)	3.19	3.85 (−2.0 to 9.7)
30 or more	1.21 (9.9)	3.10	2.41 (−4.5 to 9.3)	−4.15 (13.9)	−2.48	−3.49 (−12.5 to 5.5)
Age quit smoking (years)						
<40	−2.94 (8.9)	Ref.	Ref.	1.79 (10.8)	Ref.	Ref.
40 or older	0.46 (9.9)	2.62	3.46 (−1.3 to 8.2)	−2.82 (11.9)	−4.94	−4.44 (−10.8 to 1.9)

^1^Adjusted for the following baseline confounders: age, education, race/ethnicity, years since menopause, alcohol use, depression, and body mass index. Bonferroni corrections applied to confidence intervals to adjust for multiple comparisons.

**Table 3:** Association between smoking status and change in physical functioning and activity scores over 3 years among postmenopausal women with multiple sclerosis in the Women's Health Initiative Observational Study.

Exposure	ΔActivities of daily living (3-year baseline)			ΔPhysical activity (3-year baseline)		
	Mean change (standard deviation)	*β*-Coefficient		Mean change (standard deviation)	*β*-Coefficient	
		Crude	Adjusted^1^ (95% confidence interval)		Crude	Adjusted^1^ (95% confidence interval)
Smoking history						
Never smokers	0.01 (0.5)	Ref.	Ref.	−3.66 (12.3)	Ref.	Ref.
Ever smokers	−0.02 (0.6)	−0.03	−0.06 (−0.3 to 0.1)	−0.53 (9.8)	3.35	3.81 (0.00 to 7.6)
Former smokers	−0.01 (0.53)	0.09	0.09 (−0.2 to 0.4)	−0.60 (10.2)	−0.76	−1.06 (−7.9 to 5.7)
Current smokers	−0.10 (0.62)	−0.11	−0.14 (−0.5 to 0.2)	−0.19 (7.0)	4.00	4.72 (−2.3 to 11.7)
Age started smoking (years)						
<20	0.0 (0.6)	0.25	0.25 (−0.3 to 0.8)	−0.52 (9.3)	−1.45	−1.09 (−11.6 to 9.4)
20 to 24	−0.03 (0.4)	0.22	0.21 (−0.3 to 0.8)	−0.89 (11.3)	−2.06	−2.02 (−13.2 to 9.4)
25 or older	0.25 (0.5)	Ref.	Ref.	9.09 (12.4)	Ref.	Ref.
Cigarettes smoked (per day)						
<15	−0.04 (0.39)	Ref.	Ref.	0.14 (11.0)	Ref.	Ref.
15 or more	0.0 (0.7)	0.04	0.05 (−0.2 to 0.3)	−1.2 (8.4)	−1.80	−1.58 (−6.4 to 3.3)
Years smoked regularly						
<30	−0.08 (0.6)	Ref.	Ref.	0.04 (11.1)	Ref.	Ref.
30 or more	0.06 (0.5)	0.14	0.15 (−0.1 to 0.4)	−1.07 (6.8)	−0.92	−0.93 (−6.1 to 4.2)
Number of smoking pack- years						
<10	−0.06 (0.4)	Ref.	Ref.	−0.84 (9.6)	Ref.	Ref.
10 to 29	−0.02 (0.3)	−0.04	−0.05 (−0.3 to 0.2)	−0.96 (11.8)	2.18	2.50 (−2.7 to 7.7)
30 or more	−0.27 (1.2)	−0.29	−0.28 (−0.7 to 0.1)	−0.40 (4.6)	0.83	1.76 (−6.3 to 9.9)
Age quit smoking (years)						
<40	0.01 (0.5)	Ref.	Ref.	−0.60 (10.1)	Ref.	Ref.
40 or older	−0.10 (0.6)	−0.16	−0.15 (−0.4 to 0.1)	−1.09 (8.8)	−0.58	−0.67 (−6.2 to 4.8)

^1^Adjusted for the following baseline confounders: age, education, race/ethnicity, years since menopause, alcohol use, depression, and body mass index. Bonferroni corrections applied to confidence intervals to adjust for multiple comparisons.

**Table 4:** Association between smoking status and change in menopausal symptoms over 3 years among postmenopausal women with multiple sclerosis in the Women's Health Initiative Observational Study.

Exposure	ΔMenopausal symptoms (3-year baseline)		
	Mean change (standard deviation)	*β*-Coefficient	
		Crude	Adjusted^1^ (95% confidence interval)
Smoking history			
Never smokers	0.15 (1.5)	Ref.	Ref.
Ever smokers	0.07 (1.3)	−0.07	−0.06 (−0.5 to 0.4)
Former smokers	0.06 (1.3)	−0.12	−0.09 (−0.9 to 0.7)
Current smokers	0.17 (1.5)	0.03	0.01 (−0.8 to 0.9)
Age started smoking (years)			
<20	0.08 (1.5)	0.37	0.55 (−0.8 to 1.9)
20 to 24	0.00 (0.9)	0.25	0.37 (−1.1 to 1.8)
25 or older	−0.25 (1.4)	Ref.	Ref.
Cigarettes smoked (per day)			
<15	0.12 (1.1)	Ref.	Ref.
15 or more	0.03 (1.6)	−0.09	−0.09 (−0.7 to 0.5)
Years smoked regularly			
<30	0.03 (1.2)	Ref.	Ref.
30 or more	0.14 (1.6)	0.11	0.10 (−0.6 to 0.7)
Number of smoking pack-years			
<10	0.09 (1.4)	Ref.	Ref.
10 to 29	0.25 (1.2)	0.12	0.07 (−0.6 to 0.7)
30 or more	−0.12 (1.7)	−0.99	−0.87 (−1.9 to −0.1)
Age quit smoking (years)			
<40	0.12 (1.2)	Ref.	Ref.
40 or older	0.03 (1.4)	0.05	0.02 (−0.7 to 0.7)

^1^Adjusted for the following baseline confounders: age, education, race/ethnicity, years since menopause, alcohol use, depression, and body mass index. Bonferroni corrections applied to confidence intervals to adjust for multiple comparisons.
